# Host Cell Rab GTPases in Hepatitis B Virus Infection

**DOI:** 10.3389/fcell.2018.00154

**Published:** 2018-11-19

**Authors:** Lisa Zeyen, Reinhild Prange

**Affiliations:** Department of Virology, University Medical Center of the Johannes Gutenberg University Mainz, Mainz, Germany

**Keywords:** HBV, Rab7A, Rab33B, Rab effector, Rab GAP, virus trafficking, virus assembly, autophagy

## Abstract

Hepatitis B virus (HBV) is a leading cause of liver disease and is presently estimated to infect more than 250 million humans. The extremely successful spread of this virus among the human population is explained by its effective transmission strategies and its manifold particle types, including virions, empty envelopes and naked capsids. Due to its tiny genome, HBV depends on cellular machineries to thrive in infected hepatocytes. To enter, traverse and exit the cell, HBV exploits host membrane trafficking pathways, including intracellular highways directed by Rab GTPases. Here, we review recent discoveries focused on how HBV co-opts and perturbs host Rab GTPase functions with an emphasis on Rab7A- and Rab33B-mediated trafficking pathways. Rab7A plays bidirectional roles in the viral life cycle, as it promotes the endocytic uptake of HBV in early stages, but restricts exocytic virion release in late stages. In intermediate stages of HBV propagation, Rab33B is needed to guide the assembly of replicative progeny nucleocapsids. Rab33B acts together with its Atg5-12/16L1 effector, a protein complex required for autophagosome formation, suggesting the concept that HBV exploits this Rab/effector complex as an assembly scaffold and machine. We also discuss whether Rab-directed trafficking pathways engaged by HBV may be applicable to other virus families. Identification of overlapping Rab functions may offer new chances to develop broad-spectrum host-targeted antiviral strategies.

## Introduction

Hepatitis virus (HBV) infection remains a major public health problem. About 2 billion individuals have been infected, representing approximately 30% of world’s population, and more than 250 million remain chronically infected and have a high risk to develop cirrhosis and hepatocellular carcinoma (HCC). Despite the availability and usage of an efficient prophylactic vaccine, HBV infections continue to be an important health issue, as current therapeutics cannot cure chronic infections. Notably, HBV does not directly kill the infected liver cell, as progeny virions are released in a non-lytic manner. Instead, much of its pathogenesis is related to immune responses of the host and to its genotoxic and oncogenic potential.

**FIGURE 1:**
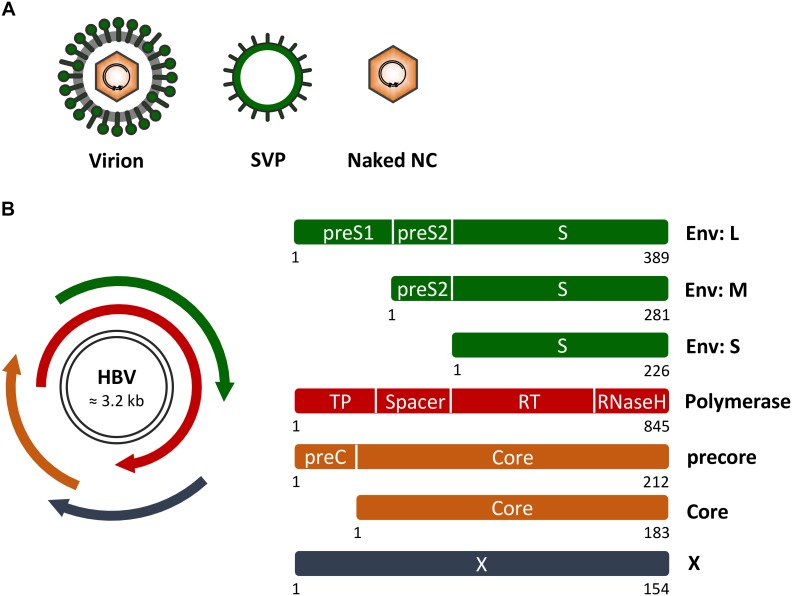
Hepatitis B virus (HBV) genome, proteins and particle types. **(A)** Schematic presentation of infectious HB virions (HBV), non-infectious empty envelope particles (SVP) and naked nucleocapids (NC). **(B)** (Left) HBV genome organization with the two black lines representing the double-stranded covalently closed circular DNA. The four overlapping open reading frames encoding the HBV envelope (Env) proteins (green), the multifunctional viral polymerase (red), the secretory precore and capsid-forming core proteins (orange), and the regulatory X protein (dark blue) are shown. (Right) Domain structures of the viral proteins. Numbers below the rectangles indicate amino acid positions as referred to HBV, genotype D. Of note, the depicted proteins are not drawn to scale.

Hepatitis B virus is an enveloped DNA virus that exclusively infects hepatocytes of humans and some non-human primates and replicates by protein-primed reverse transcription (Hu and Seeger, 2015; Li, 2015). As will be outlined in more detail below, the HBV life cycle encompasses virion attachment to liver cells, virion internalization via endocytosis, uncoating and genome delivery to the cell nucleus, transcription and translation, encapsidation of a precursor genome linked to the viral polymerase, reverse transcription, capsid maturation and envelopment, and ultimately virus egress from the host cell. With about 3 kb in size, the HBV genome is one of the smallest viral genomes known. Hence, it exhibits an extremely compact organization and contains four overlapping open reading frames (ORFs), encoding the polymerase/reverse transcriptase (RT), the capsid-forming core protein and its related secretory precore protein, three related envelope proteins and the regulatory X protein (Hu and Seeger, 2015; Figure 1). Due to its tiny genome, HBV is likely dependent on a tight interplay with host factors.

Upon infection, HBV produces at least two other particle types, subviral empty envelope particles (SVPs) and subviral naked capsid/nucleocapsid (NC) particles (Figure 1). SVPs greatly outnumber mature virions and presumably act as decoys to the immune system, as they are able to adsorb antibodies that might otherwise target viral particles (Patient et al., 2009; Prange, 2012; Blondot et al., 2016). Naked capsids/NCs can carry HBV-specific DNA and RNA species and may be instrumental in spreading infection (Bai et al., 2018). HBV exploits different intracellular trafficking routes and diverse host factors in order to release its particle types (Patient et al., 2009; Prange, 2012).

Rab proteins are key regulators of intracellular itineraries. They encompass a large group of small monomeric GTPases that organize membrane platforms and regulate vesicle budding, vesicle movement and membrane fusion. Human cells encode almost 70 Rab GTPases that perform their functions through highly regulated GTP-GDP cycles, thereby allowing the GTP-bound, active forms to recruit specific sets of effector proteins onto membranes. Each Rab protein associates with an organelle and specifies a trafficking step along endocytic, exocytic and recycling pathways (Stenmark, 2009; Wandinger-Ness and Zerial, 2014; Pfeffer, 2017).

## HBV: Infection Cycle

### HBV Uptake

Viruses use existing cell-surface receptors for attachment and exploit the endocytic network of organelles to enter the cell. For a long time, investigation of HBV has been hampered by the lack of reliable *in vitro* infection models. Historically, primary human hepatocytes (PHHs) and the human hepatoma cell line HepaRG, isolated from a donor suffering from HCC, were used to study HBV infection (Gripon et al., 2002; Hayes et al., 2016), but they are quite refractory to genetic manipulation. In a scientific breakthrough work, Yan et al. (2012) succeeded to identify the sodium taurocholate cotransporting polypeptide (NTCP) as the cellular HBV receptor. The historical challenges in establishing HBV infection systems therefore seemed likely due to a lack of adequate NTCP expression in candidate cell lines, as ectopic expression of NTCP in HuH-7 and HepG2 liver cell lines conferred susceptibility to virus infection (Yan et al., 2012; Li, 2015).

**FIGURE 2:**
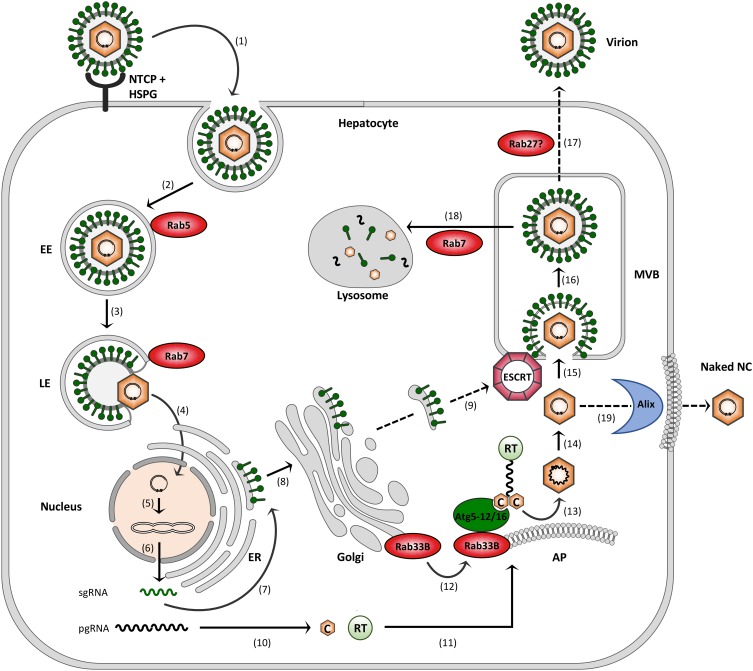
Model of the HBV infection cycle. (1) Virus attachment to HSPG and NTCP and subsequent endocytic entry, (2) Rab5-guided transport to EEs, (3) Rab7-guided transport to LEs, (4) virus uncoating and nuclear entry of NCs, (5) cccDNA formation, (6) cccDNA transcription into sgRNAs and pgRNA, (7) envelope protein synthesis at the ER, (8) envelope trafficking through the Golgi, (9) supposed envelope protein transport to MVBs, (10) core (C) and polymerase (RT) synthesis in the cytoplasm, (11) pgRNA, C and RT trafficking to NC assembly sites, (12) Rab33B/Atg5-12/Atg16L1-assisted NC assembly at autophagophores (AP), (13) NC maturation, (14) reverse transcription, (15) ESCRT-driven virus budding, (16) MVB transit, (17) supposed Rab27-mediated exosomal virus release, (18) Rab7-guided virus trafficking for lysosomal destruction, and (19) exocytosis of NCs involving the membrane-deforming capacity of Alix. Dashed arrows indicate uncovered trafficking routes.

Hepatitis B virus attachment to the hepatocyte surface involves heparan sulfate proteoglycans (HSPGs) followed by a high-affinity interaction between the myristoylated N-terminal preS region of the large (L) HBV envelope protein and NTCP (Yan et al., 2012; Sureau and Salisse, 2013; Figure 2). HBV uptake is considered to occur via endocytosis, rendering fusion of the viral envelope with internal membranes, likely to avoid envelope protein exposure on the cell surface to delay detection by immune surveillance. Endocytic pathways regulated by clathrin or caveolin-1 have been proposed for HBV uptake in PHH or differentiated HepaRG cells, respectively (Macovei et al., 2010; Huang et al., 2012). Notably, HBV internalization is escorted by cellular Rab GTPases. By using the HepaRG system combined with inducible gene knockdowns (KDs), HBV infection has been shown to require Rab5A and Rab7A, implicating that HBV is being transported from early endosomes (EE) to late endosomes (LE) (Macovei et al., 2013; Figure 2). Conversely, the KDs of Rab9 and Rab11 that are responsible for movement of endocytic vesicles to the *trans* Golgi network or the recycling endosome, respectively, had no effects, indicating that LEs are the final destination for HBV (Macovei et al., 2013). In support, an interference with the lysosomal activity by pH elevation had no effect on HBV infection, suggesting that viral uncoating takes place in a compartment preceding the lysosomes (Hayes et al., 2016).

### HBV Replication

After HBV entry and disassembly, the NCs released into the cytoplasm deliver the viral genome to the nucleus via microtubule-mediated transport mechanism. Within the nucleus, the partially double-stranded DNA (dsDNA) genome is filled-up and converted to the covalently closed circular DNA (cccDNA) (Hu and Seeger, 2015; Blondot et al., 2016; Figure 2). The episomal cccDNA persists in the nucleus as a minichromosome and is transcribed by cellular RNA polymerase II into the pregenomic RNA (pgRNA) and the viral subgenomic (sg) mRNAs. The viral RNA species are next exported to the cytoplasm where viral protein synthesis takes place. The formation of progeny virions begins with the assembly of the icosahedral NC that selectively incorporates the viral pgRNA together with the viral polymerase that is covalently linked to pgRNA via its Terminal Protein (TP) domain. The icosahedral NC is build up by 240 copies of the single core protein, consisting of 183 or 185 amino acids, depending on the genotype. The core protein contains two separate domains: the N-terminal assembly domain, which is required to form the capsid shell, and the highly basic C-terminal domain (CTD), which is essential for viral replication and mediates pgRNA/RT encapsidation (Zlotnick et al., 2015). Inside the maturing NC, the RT domain of the viral polymerase reverse transcribes the pgRNA to the dsDNA genome via an RNA/DNA hybrid intermediate (Hu and Seeger, 2015). Simultaneously, the RNA of the hybrid is degraded by the RNaseH domain of the viral polymerase. Mature progeny NCs are then enclosed by the viral envelope composed of host-derived lipids and three related viral glycoproteins: the small S, middle M and large L envelope protein that are synthesized at the endoplasmic reticulum (ER) membrane (Figure 2). Alternatively, the NCs can retransport the new dsDNA to the nucleus in order to amplify the cccDNA copy number. Notably, the cccDNA is a key obstacle for a cure of chronic hepatitis B. Current anti-HBV drugs like therapeutic nucleos(t)ide analogs can potently block reverse transcription of the pgRNA and thus virion production, but not viral transcription and viral protein expression (Nassal, 2015).

### HBV Maturation, Assembly and Release

While HBV progeny NCs mature in the cytoplasm, the envelope is build up in the secretory system. The three envelope proteins are expressed by a single ORF by the use of three different start codons and a common stop codon. Consequently, the amino acid sequence of S is repeated at the C-termini of M and L that carry the additional preS2 domain or preS1 plus preS2 domains, respectively (Prange, 2012; Blondot et al., 2016). S, M and L are synthesized as multi-spanning transmembrane proteins at the ER, where they acquire N-linked glycans. Glycan pattern profiling indicated that the envelope proteins are being exported out of the ER and transported to the Golgi complex where N-linked high mannose carbohydrates are processed (Julithe et al., 2014). Despite their similarities, the envelope proteins differ in functions, as S is the major component of the envelope and builds up its scaffold, while M is dispensable (Bruss and Ganem, 1991). Intriguingly, upon maturation L forms a dual transmembrane topology by disposing its hydrophilic N-terminal preS1+preS2 domain either at a cytosolic (i.e., corresponding to the virion inside, i-preS) or luminal (i.e., corresponding to the virion outside, e-preS) location. Both topologies of L play crucial roles in the viral life cycle, as the i-preS form recruits the mature NCs to virion budding sites, while e-preS mediates NTCP receptor binding during virus entry (Bruss, 1997; Yan et al., 2012). Notably, the S protein alone is sufficient for the production and secretion of SVPs. These defined lipoprotein complexes are formed by self-assembly of about 100 S molecules in the ER-Golgi intermediate compartment and are released via the constitutive pathway of secretion (Huovila et al., 1992).

Hepatitis B virus budding occurs at intracellular membranes and depends on functions of the endosomal sorting complexes required for transport (ESCRT) machinery (Figure 2). This machine usually accomplishes the generation of intraluminal vesicles (ILV) that bud away from the cytosol into the lumen of multivesicular bodies (MVBs). It comprises the heteromeric ESCRT-0, -I, -II and –III complexes along with the Vps4 ATPase that catalyze ILV formation in a sequential manner (Schoneberg et al., 2017). To study HBV production and egress rather than infection, transfections of hepatoma HuH-7 or HepG2 cell lines with a replication-competent HBV genome proved to be convenient model systems. These cells are refractory to infection due to low NTCP receptor levels, but allow all steps of viral replication. By means of these systems, a multitude of studies have documented the ESCRT requirements of HBV (Lambert et al., 2007; Watanabe et al., 2007; Stieler and Prange, 2014; Chou et al., 2015; Jiang et al., 2015). The ESCRT-dependency of HBV may be a hint for MVBs serving as HBV budding sites, an assumption favored by cell-imaging studies (Patient et al., 2009; Inoue et al., 2015; Yan et al., 2015). How the envelope proteins are transported from the Golgi complex to the MVBs is enigmatic (Figure 2). Given the key role of the L protein in virus assembly, it may provide the ticket to ride. L has been shown to interact with γ2-adaptin, a member of the clathrin adaptor protein family, guiding antero- and retrograde trafficking between the Golgi network and endosomes and thus potentially sorting the HBV envelope to the MVBs (Rost et al., 2006; Jurgens et al., 2013). The intracellular vesicle traffic device α-taxilin is another interaction partner of L and may control virus trafficking (Hoffmann et al., 2013). HBV is able to induce cellular autophagic processes and has been suggested to subvert the autophagy machinery for virus envelopment (Sir et al., 2010; Li et al., 2011). Hence, it seems equally possible that the HBV envelope proteins may use autophagic pathways to reach the endosomal system.

If MVBs are used as budding platform, HBV would then escape from cells via the exosomal pathway (Figure 2). Other hepatitis viruses, such as the hepatitis C virus (HCV) and hepatitis E virus (HEV) have been reported to exit infected cells via the MVB budding and transport system (Nagashima et al., 2014; Chen et al., 2015; Shrivastava et al., 2016). For exosome secretion, MVBs fuse with the plasma membrane, a process that requires Rab GTPases, particularly Rab27 family members (Ostrowski et al., 2010; Blanc and Vidal, 2018). Concordantly, the exosomal release of HCV and HEV depends on Rab27A (Nagashima et al., 2014; Chen et al., 2015; Shrivastava et al., 2016), suggesting the concept that HBV may also hijack this Rab protein to escape from cells.

## Role of Rabs in HBV Naked Capsid Release

Hepatitis B virus is an extremely successful pathogen confronting the infected host with distinct particle types that assemble and bud at specific intracellular locations. To learn whether HBV intersects with Rab-directed transport pathways, research groups initially focused on the trafficking of naked capsids/NCs whose export route as well as their functional significance are less clear. While naked capsids/NCs are rarely detected in the blood of infected patients, anti-capsid antibodies are produced in almost all patients who have been infected with HBV (Milich and McLachlan, 1986). Accordingly, capsids/NCs must exist *in vivo*, but may be quickly removed from extracellular fluids. Indeed, naked capsids/NCs carrying HBV-specific DNA and RNA species were recently detected in the blood of HBV patients as capsid-antibody-complexes (Bai et al., 2018). Since naked capsids/NCs are able to bind to cell surface-exposed HSPGs and competent for clathrin-mediated endocytosis, their uptake may enhance transmission of HBV genomes (Cooper and Shaul, 2006).

Curiously, HBV naked capsid/NC egress occurs in a non-lytic manner and generates extracellular particles devoid of a membrane lipid coat (Bardens et al., 2011). This is contrary to the non-lytic release of retroviral naked capsids (also known as Gag VLPs) that require a host-derived lipid shell during cell exit (Votteler and Sundquist, 2013). The search for dependency factors revealed that HBV naked capsid/NC release requires cellular Alix (apotosis-linked gene 2 [ALG-2]-interacting protein), a protein with multifaceted roles in membrane biology. Ectopic overexpression of Alix enhanced capsid/NC egress and mapping analyses identified its boomerang-shaped Bro1 domain to be essential and sufficient. Via its convex surface, the Bro1 domain is able to bind to membranes and may thereby account to the generation of negative curvature required for non-lytic capsid/NC budding reaction out of the cytosol (Bardens et al., 2011). Although Alix has established links to the ESCRT machinery, HBV naked capsid release does not require ESCRTs (Bardens et al., 2011; Chou et al., 2015). Remarkably, the inactivation of ESCRTs enhances the budding efficiency of naked capsids/NCs for less understood reasons. ESCRTs not only catalyze ILV formation at the MVB, as recent studies highlighted their additional role in resolving small wounds on the plasma membrane (Jimenez et al., 2014). Hence, unsealed leaky membranes due to dysfunctional ESCRT may augment naked capsid/NC egress.

To gain insights into trafficking pathways of HBV naked capsids, an RNAi screen targeting various Rab GTPases was performed in cells expressing the viral core protein only. Aside, ectopic overexpression of GTP binding-defective, dominant-negative (DN) Rab mutants was used for Rab inactivation. Thereby, the endocytic Rab7 and the exocytic Rab27 turned out to be unnecessary for capsid export (Doring and Prange, 2015). Both Rabs have links to the MVB in such that Rab7 promotes the fusion of MVBs with lysosomes, while Rab27 controls the secretion of exosomes (Ostrowski et al., 2010; Guerra and Bucci, 2016). The perturbations of either Rab7- or Rab27-guided trafficking pathways increased extracellular naked capsids yields (Doring and Prange, 2015), reminiscent for the setting observed upon ESCRT inactivation. In sum, these data implicate that HBV naked capsids apparently avoid the MVB territory along with associated ESCRT-, Rab7- and Rab27-controlled pathways to augment their extracellular release. Unlike Rab7 and Rab27, the Rab33B GTPase proved to be an HBV-dependency factor, as its KD blocked naked capsid egress (Doring and Prange, 2015).

## Role of Rab33 in Cell Physiology

The Rab33 subfamily comprises two members, Rab33A and Rab33B, who share only about 55% identity in their amino acid sequences. Beside their low homology, the expression patterns of the two genes are strikingly different. While Rab33B is ubiquitously expressed, Rab33A is preferentially expressed in the brain, in lymphocytes and melanocytes, suggesting that Rab33A and Rab33B have diverged to play independent roles in vesicular transport. Moreover, Rab33 proteins are not conserved in yeasts, *Arabidopsis thaliana*, or *Drosophila melanogaster*, indicating that Rab33 has vertebrate/mammalian-specific role(s) (Zheng et al., 1998).

Both Rab33 isoforms are resident proteins of the medial Golgi where Rab33B regulates organelle homeostasis and intra-Golgi retrograde trafficking pathways (Zheng et al., 1998; Starr et al., 2010). Retrograde transport events are instrumental for the cell to recycle glycosylation enzymes from the Golgi back to the ER and are often abused by pathogens, like certain bacterial toxins and viruses, to enter and traffic through the cell. RNAi-mediated inactivation of Rab33B inhibited the retrograde transport of the Shiga-like toxin B fragment from the *trans* to *cis* Golgi and to the ER and interfered with Golgi enzyme cycling. Conversely, the anterograde transport of a G protein variant of vesicular stomatitis virus proceeded normally in the Rab33B-depleted Golgi apparatus, underscoring the role of Rab33B in retrograde secretory traffic (Starr et al., 2010).

In addition, recent works highlighted a mechanistic link between Rab33B and the autophagic machinery. Autophagy is a eukaryotic catabolic system in which cytosolic components and organelles are sequestered by special autophagic membranes for lysosomal degradation. In mammalian cells, autophagy maintains basal homeostasis, ensures nutrient supply and also protects the cell against stresses, including microbe invasion or accumulation of aggregated proteins (Dreux and Chisari, 2010; Weidberg et al., 2011). The delivery of autophagic substrates to lysosomes occurs via double-membrane vesicular structures called autophagosomes and is executed by more than 30 specific autophagy (Atg) proteins that manage the formation and elongation of a double-membrane sack (the autophagophore), cargo capture, autophagophore closure and its final fusion with the lysosome (Geng and Klionsky, 2008; Weidberg et al., 2011). Itoh et al. (2008) discovered the intersection between the autophagy network and Rab33B by identifying Atg16L1, an essential protein in autophagophore formation, as a Rab33B-interacting protein. Notably, the association was shown to be direct and to occur in a GTP-dependent manner, hallmarks for interactions between Rab GTPases and their effectors. Mapping analysis identified a coiled-coil domain of Atg16L1 as the Rab33B binding region. The ectopic overexpression of this domain interrupted the interaction between Rab33B and wild-type Atg16L1, and importantly, suppressed autophagosome formation, indicating that Rab33B controls autophagy through Atg16L1 engagement (Itoh et al., 2008).

## Role of Rab33B in Hbv Biology

Studies that aimed to decipher the action of Rab33B in HBV naked capsid biogenesis unexpectedly showed that Rab33B primarily targets the assembly reaction of capsids and - as a consequence - their export (Doring and Prange, 2015). *De novo* capsid formation starts up in the cytoplasm of the infected hepatocyte where the monomeric core protein rapidly dimerizes, enabling the dimers to oligomerize into the icosahedral shell composed of 240 identical subunits. NC assembly is regarded to essentially mirror empty/naked capsid formation. In difference, however, the highly basic CTD domain of core interacts with the pgRNA/RT complex, cosequestering the enzyme and its template into NCs (Hu and Seeger, 2015; Zlotnick et al., 2015). According to that, it comes as little surprise that Rab33B silencing also blocked the production and release of HBV viral particles (Bartusch et al., 2017).

Biochemical and cell imaging studies of HBV-replicating HuH-7 cells indicated that the silencing of Rab33B substantially impaired the synthesis, assembly and/or stability of core/capsids concomitant with improper NC formation and trafficking to budding sites (Bartusch et al., 2017). As HBV capsid assembly can be recapitulated in bacteria and cell-free systems, it had largely been considered as a spontaneously occurring self-assembly process, only regulated by the presence of capsid protein subunits themselves (Zlotnick et al., 2015). The crowded cytoplasm of the hepatocyte, however, may be an unfavorable environment for homotypic core interactions. In support, recent reports demonstrated that HBV capsid assembly is assisted by host factors, like chaperones and chaperone-like proteins, in hepatocytes (Chen et al., 2011; Seo et al., 2018). Moreover, when HBV capsid assembly was analyzed in wheat germ extracts, it turned out to be sensitive against detergents, implicating that intact membrane surfaces may be involved (Lingappa et al., 2005). Studies of the underlying mechanisms led to the identification of a hitherto unknown membrane-binding motif in the highly basic CTD domain of core (Bartusch et al., 2017). Hence, the HBV core appears to bypass the constraints of crowded cytoplasm by routing capsid assembly to membranes in order to increase the local concentration of dimeric core proteins. Importantly, deficient Rab33B reduced the membrane association of core thereby provoking aberrant core/capsid accumulations that are prone to degradation (Bartusch et al., 2017). Whether the HBV envelope may be an additional client of Rab33B remains to be determined. As outlined above, cellular γ2-adaptin interacts with the HBV L envelope protein in a productive manner (Rost et al., 2006). γ2-Adaptin in turn is able to associate with rabaptin-5 (also known as RABEP1), a known effector protein of the endosomal Rab5 GTPase and of Rab33B (Stenmark et al., 1995; Mattera et al., 2003; Pusapati et al., 2012). Albeit being undetermined, a putative super-complex composed of Rab33B-rabaptin-5-Rab5 along with associated γ2-adaptin/L may escort HBV envelope protein trafficking out of the Golgi to early endosomes and ultimately to MVBs.

The proviral role of Rab33B is substantiated by its transcriptional upregulation in HBV-replicating cells (Bartusch et al., 2017). Intriguingly, HCV has also been reported to enhance Rab33B expression. Host gene expression profiling of HCV-replicating HuH-7.5 cells demonstrated elevated Rab33B-specific transcript levels (Blackham et al., 2010). Gain of function analysis verified that HCV secretion depends on Rab33B (Blackham et al., 2010; Mankouri et al., 2016), indicating that both hepatotropic viruses, HBV and HCV, require Rab33B activity. With regard to virus-induced modulations of host gene expression, it should be noted that Rab18 is another Rab GTPase that is upregulated by HBV and HCV. In the case of HBV, elevated Rab18 levels were found in virus-replicating hepatoma cells and clinical HCC tissues (You et al., 2013). Since Rab18 is a key coordinator of lipogenesis, the HBV-induced upregulation of Rab18 promoted abnormal lipogenesis and dysregulated proliferation thereby enhancing HCC progression (You et al., 2013). To manage lipid storage and mobilization, Rab18 is a resident of lipid droplets (LDs) that are hijacked by HCV as assembly platforms. As Rab18 is essential for HCV core protein trafficking to LDs, the virus appears to profit from increased Rab18 levels (Mannova et al., 2006; Salloum et al., 2013).

## Role of Rab33B Effectors in HBV Biology

**FIGURE 3:**
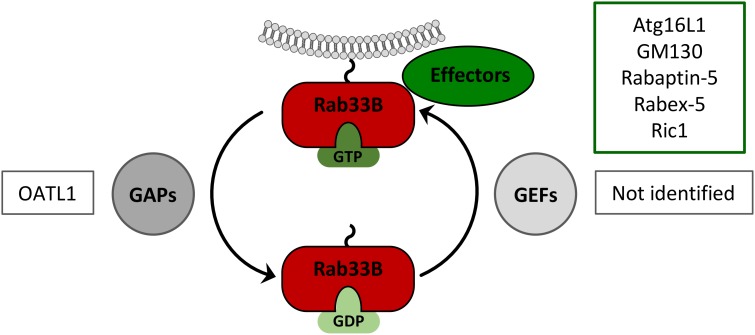
Rab33B GTPase cycle. Rab33B associates with membranes via its C-terminal isoprenoid moiety and switches between GDP- and GTP-bound forms thereby adopting different conformations. Conversion from the GDP- to the GTP-bound form is catalyzed by GDP/GTP exchange factors (GEFs). Conversion from the GTP- to the GDP-bound forms occurs by GTP hydrolysis, facilitated by GTPase-activating proteins (GAPs). The active GTP-bound form of Rab33B is able to interact with effector molecules. Known Rab33B-specific effectors and GAPs are indicated in the corresponding boxes.

Rab proteins perform their functions through tightly controlled GTP-GDP cycles, thereby allowing the GTP-bound, active forms to interact with specific effector proteins (Figure 3). In HBV-replicating cells, the ectopic expression of a GDP-restricted Rab33B mutant phenocopied the effects of deficient Rab33B, implicating that effectors may be involved (Bartusch et al., 2017). Thus far, rabaptin-5, GM130 (also known as GOLGA2), Ric1 and Atg16L1 have been described as Rab33B effectors (Valsdottir et al., 2001; Itoh et al., 2008; Pusapati et al., 2012). Considering that HBV and Rab33B both have links to the autophagy machinery, Atg16L1 appears to be one prime effector in Rab33B-assisted HBV assembly.

Atg16L1 comprises one component of two ubiquitin-like (UBL) conjugation systems that are recruited to the forming autophagophore and mediate its expansion. In one system, members of the LC3 (microtubule associated protein 1 light chain 3) family are firstly cleaved by Atg4 to expose their C-terminal cysteines. Analogous to ubiquitination, LC3 is next conjugated to the membrane lipid phosphatidylethanolamine (PE) by Atg7 and Atg3, that act as E1-like protein and E2-like enzymes, respectively. In the other UBL system, the ubiquitin-like protein Atg12 is covalently conjugated to Atg5 that depends on the catalytic activity of Atg7 (E1-like) and Atg10 (E2-like). The Atg5-12 conjugate associates non-covalently with Atg16L1 and the resulting complex scaffolds the maturing autophagophore. Moreover, the Atg5-12/16L1 complex acts as an E3-like protein during the LC3 lipidation reaction, as it facilitates the recruitment of the Atg7/Atg3 proteins (Geng and Klionsky, 2008; Weidberg et al., 2011). Co-depletion analyses confirmed that the Atg5-12/16L1 complex firmly interacts with Rab33B, as Rab33B silencing triggered the concomitant downregulation of Atg5-12 and Atg16L1 (Bartusch et al., 2017).

Extensive RNAi analysis performed in HBV-replicating HuH-7 cells and in cells expressing only the HBV core protein demonstrated that silencing of Atg5, Atg12 and Atg16L1 interfered with viral capsid/NC formation and/or stability and strongly diminished virus particle and naked capsids yields (Doring and Prange, 2015; Doring et al., 2018). Collaterally, the association of core/NC with membranes and their sorting to envelope-positive compartments were compromised, a phenotype closely mirroring that of Rab33B KD cells. HBV was found to be strictly dependent on Atg12 conjugated to Atg5, as unconjugated Atg5 failed to rescue the block in virus propagation. In support of this, silencing of Atg7 and Atg10, enzymes that are essential for Atg5-12 conjugation, also impaired virus production (Sir et al., 2010; Doring et al., 2018). HBV gained access to the Atg5-12/16L1 effector through binding of its core protein with the Atg12 subunit. Worth mentioning, core interacts with the so-called intrinsically disordered region (IDR) of Atg12 that is not essential for normal autophagy (Doring et al., 2018). Hence, interfering the HBV/Atg12 interplay may be a new tool for virus control. Conceivably, cell-penetrating peptides mimicking the IDR of Atg12 may out-compete the interaction between HBV core and full-length Atg12 thereby blocking virus production without affecting cellular autophagy.

These data suggest that HBV may coopt the Atg5-12/16L1 complex, tethered to the expanding autophagophore membrane, as a scaffold for capsid/NC assembly. Simultaneously, the Atg16L1 moiety may recruit Rab33B-decorated, Golgi-derived vesicles in order to supply lipids for the growing autophagophore (Figure 2). Mechanistically, this scenario is similar to retroviral Gag assembly that occurs at the plasma membrane (PM) of infected cells. In case of the human immunodeficiency virus type 1 (HIV-1), Rab27A has been shown to influence virus assembly via effects on phosphatidylinositol 4,5-bisphosphate (PI(4,5)P_2_), a phosphoinositide interacting with the viral Gag. Rab27A KD impaired HIV-1 assembly due to an impaired Rab27A-guided trafficking of endosomes to the PM concomitant with reduced membrane (PI(4,5)P_2_) delivery to interact with Gag (Gerber et al., 2015). Beside HBV, a couple of other viruses including coxsackievirus, dengue virus, influenza A virus and HCV have engineered strategies to coopt autophagic vesicles as platforms for replication (Dreux and Chisari, 2010; Jackson, 2014; Shrivastava et al., 2016). For example, HCV has been shown to evoke rearrangements of intracellular membranes, the membrane web (MW) structures, in which viral replication and assembly take place. MW formation was shown to require the Atg5-12/16L1 conjugate, establishing the HCV replication scaffold (Fahmy and Labonte, 2017). Interestingly, both viruses – HBV and HCV – were shown to require early, non-degradative stages of autophagy, while late stages were not mandatory, as evidenced by their LC3- and Atg4B-independencies (Fahmy and Labonte, 2017; Doring et al., 2018). This may explain why both viruses induce autophagy without ending up in the destructive autophagolysosomes.

## Role of Rab33B Gaps in HBV Biology

GTPase-activating proteins are GTPase accelerating proteins that bind to the target Rab protein to stimulate the hydrolysis of the bound GTP to GDP and thereby reconverting the Rab back to its inactive state (Figure 3). All of the characterized Rab GAP proteins so far contain a conserved TBC (Tre2/Bub2/Cdc16) domain that is required for their activity (Muller and Goody, 2018). Upon screening of a mammalian TBC protein library, ornithine aminotransferase-like 1 protein (OATL1), also known as TBC1D25, was identified as a Rab33B-specific GAP (Itoh et al., 2011). The overexpression of OATL1 was shown to delay the maturation of autophagosomes, implicating that OATL1 is directed to autophagosomes to inactivate Rab33B, bound to the Atg5-12/16L1 complex (Itoh et al., 2011). Vice versa, OATL1 silencing is considered to keep Rab33B in its catalytic active form. In line with this, OATL1 silencing increased HBV production, likely to be due to over-active Rab33B (Bartusch, 2018). Contrary to GAPs, guanine nucleotide exchange factors (GEFs) catalyze the conversion of the GDP-bound Rab into the GTP-bound, active form. Since Rab33B-specific GEFs have not been identified so far, their possible roles in the HBV life cycle are unknown.

## Role of Rab7 in Cell Physiology

In mammals, there are two Rab7 proteins, Rab7A (also referred to as Rab7) and Rab7B, with a limited identity of about 50%. Both Rab7 proteins are localized to late endosomes and lysosomes and are key organizers of endo-lysosomal membrane trafficking processes (Wandinger-Ness and Zerial, 2014; Guerra and Bucci, 2016). Aside, Rab7B is also involved in late endosome-to-Golgi retrograde transport, as exemplified by its need to guide trafficking of the cholera toxin B subunit from endosomes to the Golgi (Guerra and Bucci, 2016). Rab7A plays a key role in the maturation of late endosomes/MVBs and autophagosomes, guiding the transportation of vesicle cargos along microtubules and accomplishing the fusion step with lysosomes. On the endosomal membrane activated Rab7A binds to RILP (Rab-interacting lysosomal protein), a proven effector of Rab7, that in turn forms a complex with dynein–dynactin to guide the inward transport of endosomes to the minus-end of microtubules (Guerra and Bucci, 2016). The pleckstrin homology domain containing protein family member 1 (PLEKHM1) is another effector of Rab7A that together participates in the endosome-lysosome fusion step (McEwan et al., 2015).

## Role of Rab7A in HBV Biology

During HBV entry, Rab7A has been shown to act as a dependency factor (Macovei et al., 2013). Conversely, in the late steps of HBV replication Rab7A seemingly restricts virus propagation, as shown in three independent reports. By using stably virus-expressing HepG2.2.15 cell lines, Inoue et al. (2015) demonstrated that HBV induces pronounced tubular networks that trigger fusion events of MVBs, autophagosomes and lysosomes and promote the degradation of HBV due to activation of Rab7A. Rab7A KD augmented HBV release, likely due to a reduced delivery of the virus to the degrading lysosome (Figure 2). The expression of a GDP-restricted Rab7A mutant phenocopied the effect of Rab7A silencing, indicative for an involvement of effector proteins. By defining the HBV component responsible for Rab7A activation, the authors uncovered the precore protein to be sufficient. HBV might thus decelerate its own release through precore-mediated Rab7A stimulation to decrease the immune response of the infected host.

Consistent with this, Lin et al. (2018) reported that Rab7A silencing increased HBV production in HepG2.2.15 cells. The overexpression of active Rab7A decreased HBV propagation, while expression of inactive Rab7A had opposite effects, adding further proof that Rab7A activation reduces viral loads. The KD of the Rab7A effector PLEKHM1 strongly increased HBV yields, indicating that hampered endo-lysosomal fusion events benefit HBV production (Lin et al., 2018). In addition, Lin et al. (2018) and Zhou et al. (2016) demonstrated that the presence of HBV reduced the expression of Rab7A in hepatoma cells, likely to prevent its own clearance in degradative pathways.

As mentioned above, HCV is another hepatotropic virus that abuses autophagy functions for maturation but avoids autophagosomal/lysosomal destruction. Like HBV, HCV has been shown to block Rab7A-mediated endosome-lysosome fusion steps upon *in vitro* replication in HuH-7.5 cell lines (Wozniak et al., 2016). However, unlike HBV, HCV infection did not change Rab7A quantity or activation state. Instead, HCV triggered cleavage of RILP, a Rab7A effector responsible for linking Rab7A vesicles to dynein motor complexes. RILP cleavage promotes the association of Rab7A-decorated vesicles with kinesin rather than dynein facilitating the outward more than the inward movement of vesicles and thus the secretion of HCV (Wozniak et al., 2016). Hence, the destruction of an effector of Rab7A is yet another intriguing strategy by which a virus modifies the host cell to optimize virion secretion.

## Concluding Remarks

Hepatitis B virus is an obligate intracellular pathogen charging the infected hepatocyte with a huge amount of particle types. For particle biogenesis and release, HBV apparently evolved to utilize intracellular trafficking pathways directed by host cell Rab GTPases. The interactions depicted herein are first examples of a likely much more comprehensive phenomenon of HBV intersections with Rab-regulated trafficking routes. For example, Rab control is likely to be involved in HBV envelope trafficking within and out of the secretory system. Despite there is conclusive evidence for the role of ESCRTs in the virus budding reaction, the budding site of HBV and its pathway of release have not been unambiguously defined. Inspection of roles of exocytic Rabs, like Rab27, Rab35 and Rab37, may provide answers to these questions. As exemplified for the Rab33B GTPase, HBV gains access to the cellular Rab network via exploitation of Rab-specific effector proteins, an access mode possibly shared by other viruses. In this context, it will be also of interest to know whether the conjoint Rab33B/Atg5-12/16L1 complex, co-opted by HBV, may play a common role in autophagy-assisted virus assembly reactions. New knowledge of viral interactions with Rab GTPases and their effectors will provide deeper insights of the HBV life cycle that will lead to more effective antivirals.

## Author Contributions

Both authors had made substantial, direct, and intellectual contribution to the work and approved it for publication.

## Conflict of Interest Statement

The authors declare that the research was conducted in the absence of any commercial or financial relationships that could be construed as a potential conflict of interest.
